# Lobectomy versus segmentectomy in patients with stage T (> 2 cm and ≤ 3 cm) N0M0 non-small cell lung cancer: a propensity score matching study

**DOI:** 10.1186/s13019-022-01867-x

**Published:** 2022-05-11

**Authors:** Linlin Wang, Lihui Ge, Sibo You, Yongyu Liu, Yi Ren

**Affiliations:** 1 Department of Thoracic Surgery, Shenyang Chest Hospital and Tenth People’s Hospital, No. 11, Beihai Street, Dadong District, Shenyang, 110044 Liaoning People’s Republic of China; 2 Department of Health Management, Shengjing Hospital of China Medical University, No. 36, Sanhao Street, HePing District, Shenyang, 110004 Liaoning People’s Republic of China

**Keywords:** Forest plots, Non-small cell lung cancer (NSCLC), Propensity score matching (PSM), Surveillance, Epidemiology, and End Results (SEER) database, Survival analysis

## Abstract

**Background:**

The safety and effectiveness of lung segmentectomy in patients with early non-small cell lung cancer (NSCLC) remains controversial. We have therefore reviewed the clinicopathologic characteristics and survival outcomes of patients treated with lobectomy or segmentectomy for early T (> 2 and ≤ 3 cm) N0M0 NSCLC.

**Methods:**

We obtained data from the Surveillance, Epidemiology, and End Results database for patients who underwent lobectomy or segmentectomy between 2004 and 2015. To reduce bias and imbalances between the treatment groups, propensity score matching analysis was performed. We used Kaplan–Meier curves to estimate overall survival (OS) and lung cancer-specific survival (LCSS). We conducted univariate and multivariate Cox proportional hazards regression analyses to identify independent prognostic factors for OS and cancer-specific survival, and applied the Cox proportional hazards model to create forest plots.

**Results:**

Before matching, both univariate and multivariate Cox regression analyses revealed that patients who underwent lobectomy exhibited better OS (*P* < 0.001) and LCSS (*P* = 0.001) than patients who underwent segmentectomy. However, after matching, survival differences between the groups were not significant; OS (*P* = 0.434) and LCSS (*P* = 0.593). Regression analyses revealed that age and tumor grade were independent predictors of OS and LCSS (*P* < 0.05).

**Conclusions:**

Patients with stage T (> 2 and ≤ 3 cm) N0M0 NSCLC undergoing segmentectomy can obtain OS and LCSS similar to those obtained with lobectomy. Further studies are required considering the solid component effects and pathologic tumor types regarding segmentectomies. Additional long-term survival and outcome analyses should be conducted with larger cohorts.

## Introduction

Lung cancer accounts for 11.6% of all cancers and 18.4% of cancer deaths. Malignant tumors are associated with the highest morbidity and mortality rates [1]. The most recent estimate predicts 228,820 new cases and 135,720 deaths in 2020, demonstrating the tremendous global impact of this disease, which has a 5-year survival rate of approximately 19% [2]. Surgery is the preferred treatment for early-stage non-small cell lung cancer (NSCLC), and it is also the only proven method to cure lung cancer [3, 4]. The current National Comprehensive Cancer Network (NCCN) guidelines recommend lobectomy as the first-line treatment for early NSCLC. High-resolution computed tomography has increased the detection rate of early lung cancer. Compared with traditional lobectomy, segmentectomy fulfills the oncological requirements and also reduces some loss of lung function [5]. However, there is controversy regarding whether segmental resection is more appropriate than lobectomy for surgical treatment of early NSCLC [6]. This study aimed to evaluate the clinicopathologic characteristics and survival outcomes of patients with NSCLC after segmentectomy compared to those after lobectomy. We used a population-based national registry, the Surveillance, Epidemiology, and End Results (SEER) database, to analyze the clinical characteristics and prognoses of patients with T (> 2 and ≤ 3 cm) N0M0 NSCLC who received either segmentectomy or lobectomy. Based on the survival analysis results, we created forest plots using the Cox proportional hazards model.

## Methods

### Data collection

**Fig. 1:**
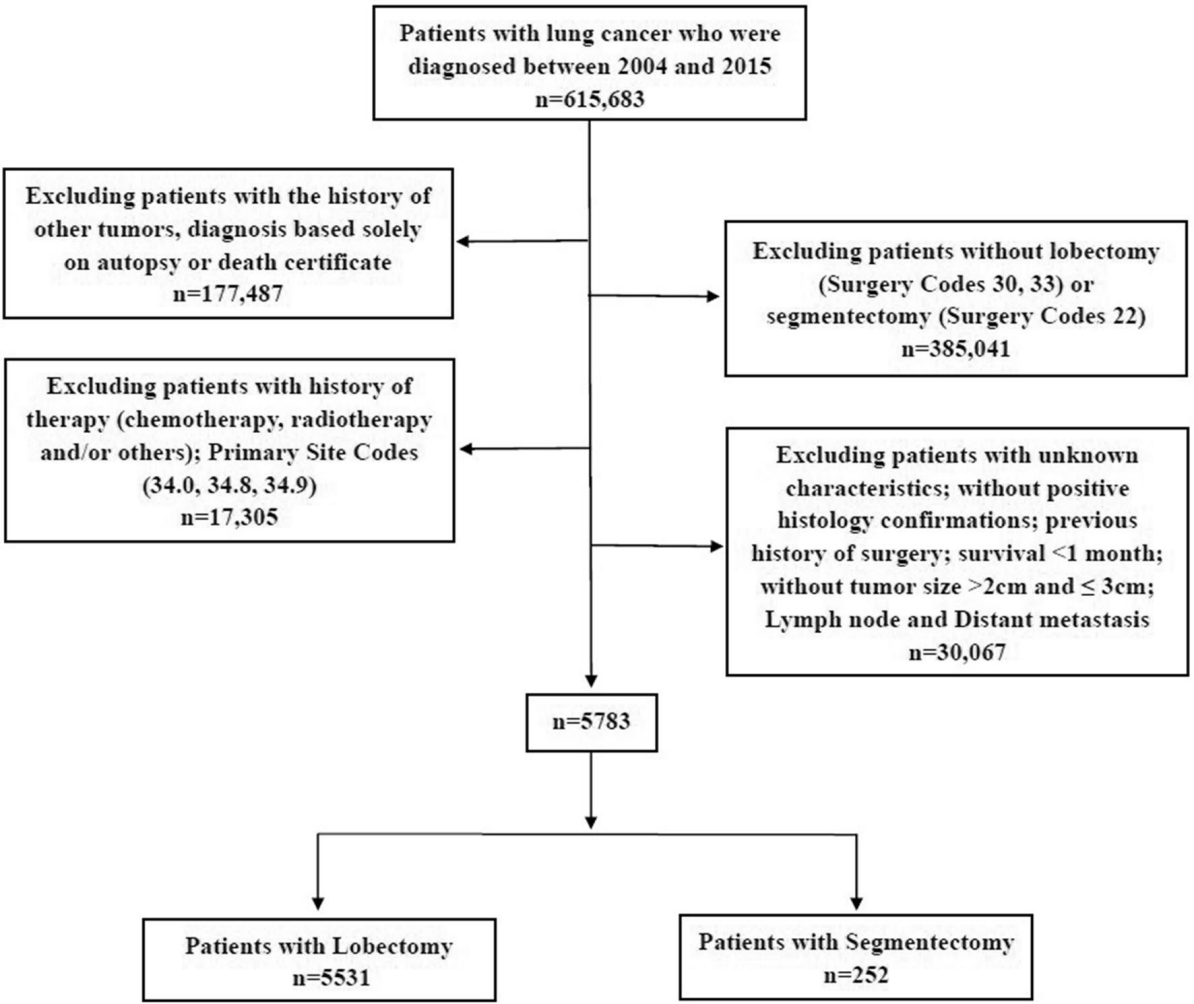
Flow chart showing selection of patients with early non-small cell lung cancer

We extracted data from the SEER database (https://seer.cancer.gov/) using SEER*Stat software (v8.3.6, https://seer.cancer.gov/seerstat/) to identify patients with a confirmed diagnosis of NSCLC between 2004 and 2015 undergoing segmentectomy (SEER Surgery Code: 22) or lobectomy (SEER Surgery Codes: 30, 33). The inclusion criteria were: (1) diagnosis between 2004 and 2015; (2) tumor size (TS, maximum diameter on pathological assessment) > 2 cm, and ≤ 3 cm; (3) NSCLC diagnosis confirmed on histology; (4) one primary tumor; (5) survival for at least 1 month; (6) active follow-up; and (7) available clinical information. The exclusion criteria were: (1) incomplete survival or clinical data, including unknown race, tumor grade, marital status, SEER cause-specific death classifications, and vital status recodes; (2) previous history of surgery; (3) history of therapy (chemotherapy, radiotherapy and/or others); (4) diagnosis based solely on autopsy or death certificate (Fig. 1). The institutional review board of Shenyang Chest Hospital & Tenth People's Hospital approved the study. All methods were performed in accordance with the relevant guidelines and regulations.

### Variables

This study utilized public data from the SEER database. The covariates included age, sex, race, marital status, laterality, primary site, histopathology, and tumor grade. We classified age into four groups: ≤ 60, 61–70, 71–80, and ≥ 81. Laterality was defined as left and right, with the primary site classified as upper, middle, and lower. The histopathology was defined as adenocarcinoma (ADC), squamous cell carcinoma (SCC), and other tumor types (others). The grade was classified as well- (I), moderately- (II), and poorly differentiated, or undifferentiated (III–IV), based on the eighth edition of the American Joint Committee on Cancer lung cancer staging system, and updated TS (> 2 and ≤ 3 cm) for all patients over all time periods. OS was defined as the time from operative day to death from any cause or lost to follow-up. LCSS was defined as the time from diagnosis to lung cancer, excluding other causes of death.

### Propensity score matching

Propensity score matching (PSM) reduces the effects of bias and confounding variables, by removing confounding factors between groups, thereby increasing comparability between the groups [7]. We used PSM to control for inherent biases associated with cohort studies. Propensity scores were derived by logistic regression based on potentially confounding baseline characteristics of patients, including age, sex, race, marital status, laterality, primary site, histopathology, tumor grade, SEER cause-specific death classification and vital status recode. Subsequently, patients who underwent segmentectomy and lobectomy were paired, using the nearest neighbor matching method, a caliper width of 0.2, and no replacement, resulting in a 1:2 matched sample, reducing standardized differences to < 0.1 after matching. Continuous and categorical variables were compared using the Student’s *t* test, chi-square test, and analysis of variance (ANOVA) after matching. This study employed Cox regression after PSM to identify more reliable causal inferences.

### Statistical analysis

In this study, categorical variables are expressed as percentages, and continuous variables are expressed as means ± standard deviations (SDs). Variables were compared using the Student’s *t* test, chi-square test, and ANOVA. We used the Kaplan–Meier method to generate survival curves and analyzed differences between curves using the log-rank test. We used the Cox proportional hazards model to examine independent prognostic factors and calculate the hazard ratio [HR] and corresponding 95% confidence interval [CI]. Specific results are depicted as forest plots. Power Analysis and Sample Size (PASS) software was used for sample evaluation. Data were analyzed with Statistical Product and Service Solutions 26.0 software (SPSS, Inc., Chicago, IL, USA). *P*-values < 0.05 (two-sided) were considered statistically significant. Survival curves and the forest plot were drawn with GraphPad Prism software (Version 8.3.1, GraphPad software Inc, California, USA).

## Results

### Patient and clinicopathologic characteristics

A total of 5783 patients who underwent segmentectomy or lobectomy between 2004 and 2015 were selected from the SEER database. Of these, 5531 (95.64%) received lobectomies, and 252 (4.36%) received segmentectomies. The patient characteristics are shown in Table 1. The two groups were similar regarding sex, race, marital status, primary tumor site, histopathology and tumor grade.

**Table 1:** Baseline patient characteristics before and after propensity score matching

Characteristic	Surgical procedure unmatching				Surgical procedure matching			
	Total (N = 5783)	Lobectomy (N = 5531)	Segmentectomy (N = 252)	*P*^b^ value	Total (N = 755)	Lobectomy (N = 503)	Segmentectomy (N = 252)	*P* value
Age (years), n (%)				0.001				0.472
≤ 60	1351 (23.4)	1308 (23.6)	43 (17.1)		129 (17.1)	86 (17.1)	43 (17.1)	
61–70	2011 (34.8)	1928 (34.9)	83 (32.9)		238 (31.5)	155 (30.8)	83 (32.9)	
71–80	1945 (33.6)	1855 (33.5)	90 (35.7)		295 (39.1)	205 (40.8)	90 (35.7)	
≥ 81	476 (8.2)	440 (8.0)	36 (14.3)		93 (12.3)	57 (11.3)	36 (14.3)	
Mean ± SD	67.47 ± 10.25	67.37 ± 10.24	69.75 ± 10.32	< 0.001	69.67 ± 9.96	69.63 ± 9.78	69.75 ± 10.32	0.880
Sex, n (%)				0.743				0.316
Female	3225 (55.8)	3087 (55.8)	138 (54.8)		394 (52.2)	256 (50.9)	138 (54.8)	
Male	2558 (44.2)	2444 (44.2)	114 (45.2)		361 (47.8)	247 (49.1)	114 (45.2)	
Race, n (%)				0.605				0.944
White	4871 (84.2)	4664 (84.3)	207 (82.1)		625 (82.8)	418 (83.1)	207 (82.1)	
Black	463 (8.0)	439 (7.9)	24 (9.5)		70 (9.3)	46 (9.1)	24 (9.5)	
Others	449 (7.8)	428 (7.7)	21 (8.3)		60 (7.9)	39 (7.8)	21 (8.3)	
Marital status, n (%)				0.397				0.859
No^a^	3430 (59.3)	3237 (59.4)	143 (56.7)		425 (56.3)	282 (56.1)	143 (56.7)	
Yes	2353 (40.7)	2244 (40.6)	109 (43.3)		330 (43.7)	221 (43.9)	109 (43.3)	
Laterality, n (%)				0.005				0.817
Left	2418 (41.8)	2291 (41.4)	127 (50.4)		376 (49.8)	249 (49.5)	127 (50.4)	
Right	3365 (58.2)	3240 (58.6)	125 (49.6)		379 (50.2)	254 (50.5)	125 (49.6)	
Primary Site, n (%)				0.063				0.659
Upper	3524 (60.9)	3380 (61.6)	144 (57.1)		428 (56.7)	284 (56.5)	144 (57.1)	
Middle	305 (5.3)	297 (5.4)	8 (3.2)		31 (4.1)	23 (4.6)	8 (3.2)	
Lower	1954 (33.8)	1854 (33.5)	100 (39.7)		296 (39.2)	196 (39.0)	100 (39.7)	
Histopathology, n (%)				0.111				0.290
ADC	2450 (42.4)	2355 (42.6)	95 (37.7)		282 (37.4)	187 (37.2)	95 (37.7)	
SCC	1234 (21.3)	1184 (21.4)	50 (19.8)		174 (23.0)	124 (24.7)	50 (19.8)	
Others	2099 (36.3)	1992 (36.0)	107 (42.5)		299 (39.6)	192 (38.2)	107 (42.5)	
Grade, n (%)				0.227				0.917
I	1357 (23.5)	1309 (23.7)	48 (19.0)		140 (18.5)	92 (18.3)	48 (19.0)	
II	2649 (45.8)	2529 (45.7)	120 (47.6)		356 (47.2)	236 (46.9)	120 (47.6)	
III–IV	1777 (30.7)	1693 (30.6)	84 (33.4)		259 (34.3)	175 (34.8)	84 (33.3)	
OS, n (%)				0.001				0.739
Alive	3754 (64.9)	3616 (65.4)	138 (54.8)		407 (53.9)	269 (53.5)	138 (54.8)	
Dead	2029 (35.1)	1915 (34.6)	114 (45.2)		348 (46.1)	234 (46.5)	114 (45.2)	
LCSS, n (%)				0.020				0.755
Alive	4706 (81.4)	4515 (81.6)	191 (75.8)		567 (75.1)	376 (74.8)	191 (75.8)	
Dead	1077 (18.6)	1016 (18.4)	61 (24.2)		188 (24.9)	127 (25.2)	61 (24.2)	

ADC, adenocarcinoma; SCC, squamous cell carcinoma; I, well differentiated; II, moderately differentiated; III-IV, poorly differentiated/ undifferentiated; OS, overall survival; LCSS, lung cancer-specific survival; SD, standard deviation

^a^No included separated, single (never married), divorced, unmarried or domestic partner and widowed

^b^*P* value between lobectomy and segmentectomy was calculated using the chi-square test

### Survival analyses

**Fig. 2:**
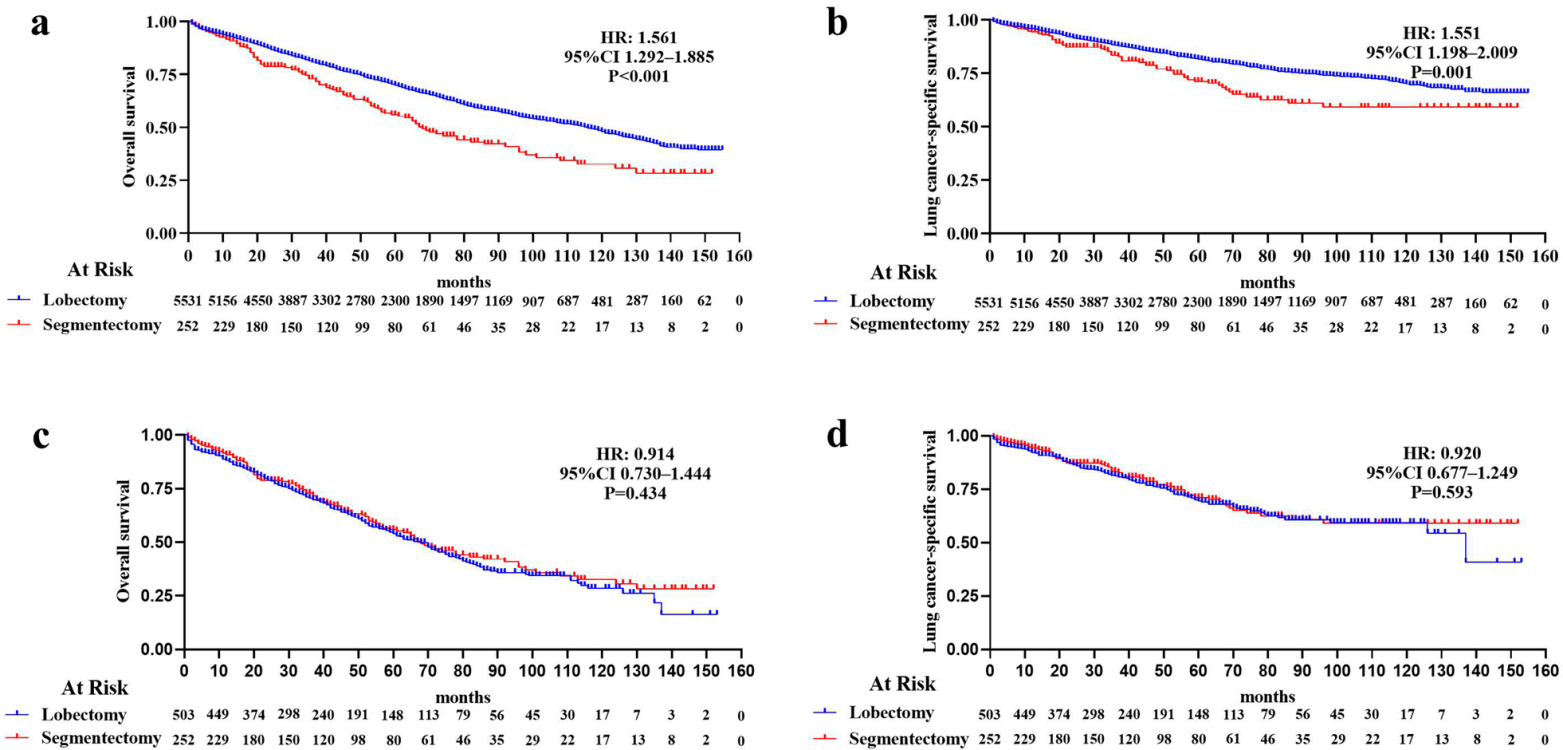
(Top) Kaplan–Meier survival curves for overall survival (OS) in early non-small cell lung cancer (NSCLC) patients after lobectomy and segmentectomy before propensity score matching **a** OS, hazard ratio (HR) 1.561; 95% confidence interval (CI) 1.292–1.885; *P* < 0.001; **b** lung cancer-specific survival (LCSS), HR 1.551; 95% CI 1.198–2.009; *P* = 0.001. (Bottom) Kaplan–Meier survival curves for OS in patients receiving segmentectomies and lobectomies after propensity score matching. **c** OS, HR 0.914; 95% CI 0.730–1.444; *P* = 0.434; **d** LCSS, HR 0.920; 95% CI 0.677–1.249; *P* = 0.593

Among the 5783 patients, the mean follow-up was 56.57 ± 38.31 (lobectomy: 56.97 ± 38.32, segmentectomy: 47.72 ± 37.03) months; *P* < 0.001. The median OS was 116 (95% CI 109.74–122.26) months for lobectomy *vs.* 68 (95% CI 56.39–79.61) months for segmentectomy. The 1-, 3-, 5-, and 10-year OS rates for all patients were 92.9, 80.4, 69.4, and 47.3%, respectively. For patients receiving lobectomies and those receiving segmentectomies the 1-, 3-, 5-, and 10-year OS rates were 92.9, 80.7, 69.6, and 48.0%; and 90.8, 72.8, 55.2, and 30.7%, respectively. Both OS (HR 1.561; 95% CI 1.292–1.885; *P* < 0.001) and LCSS (HR 1.551; 95% CI 1.198–2.009; *P* = 0.001) were significantly worse for patients receiving segmentectomies compared with those receiving lobectomies (Fig. 2a, b).

We used univariate analyses to identify possible prognostic factors for lobectomy or segmentectomy for treating patients with NSCLC. We identified statistically significant (*P* < 0.05) correlations between OS and LCSS with surgical procedure, age, sex, race, marital status, histopathology and grade (Table 2). Laterality and primary site were not significant prognostic factors in our univariate analyses (*P* > 0.05). For OS, patients receiving lobectomies were significantly different compared with patients receiving segmentectomies (*P* < 0.05), regarding age > 60 years; sex; white, or other race; marital status; a right lateral; tumor location; ADC, or another tumor type; and grade I–III/IV tumors. For LCSS, the parameters showing significant differences between patients receiving lobectomies *vs*. segmentectomies (*P* < 0.05) were age ≥ 61 but ≤ 70 years and age ≥ 81 years; female sex; white, or other race; married; a right lateral or lower tumor location; ADC, and grade I tumors.

**Table 2:** Univariate and multivariate analyses of OS and LCSS before propensity score matching

Characteristic	Univariate analysis (unmatching)				Multivariate analysis (unmatching)			
	OS		LCSS		OS		LCSS	
	HR (95% CI)	*P* value	HR (95% CI)	*P* value	HR (95% CI)	*P* value	HR (95% CI)	*P* value
Surgical procedure								
Lobectomy	Reference		Reference		Reference		Reference	
Segmentectomy	1.561 (1.292–1.885)	< 0.001	1.551 (1.198–2.009)	0.001	1.447 (1.197–1.750)	< 0.001	1.436 (1.108–1.861)	0.006
Age (years)								
≤ 60	Reference		Reference		Reference		Reference	
61–70	1.599 (1.390–1.838)	< 0.001	1.371 (1.147–1.638)	0.001	1.484 (1.290–1.708)	< 0.001	1.307 (1.093–1.564)	0.003
71–80	2.314 (2.023–2.648)	< 0.001	1.780 (1.497–2.118)	< 0.001	2.189 (1.911–2.507)	< 0.001	1.722 (1.445–2.052)	< 0.001
≥ 81	3.870 (3.281–4.565)	< 0.001	2.469 (1.962–3.107)	< 0.001	3.674 (3.110–4.340)	< 0.001	2.406 (1.908–3.035)	< 0.001
Sex								
Female	Reference		Reference		Reference		Reference	
Male	1.525 (1.398–1.664)	< 0.001	1.378 (1.223–1.553)	< 0.001	1.502 (1.370–1.647)	< 0.001	1.320 (1.165–1.496)	< 0.001
Race								
White	1.619 (1.322–1.982)	< 0.001	1.373 (1.060–1.779)	0.016	1.544 (1.260–1.892)	< 0.001	1.302 (1.003–1.689)	0.047
Black	1.671 (1.300–2.149)	< 0.001	1.615 (1.171–2.228)	0.003	1.697 (1.316–2.188)	< 0.001	1.563 (1.129–2.164)	0.007
Others	Reference		Reference		Reference		Reference	
Marital status								
No	0.790 (0.724–0.862)	< 0.001	0.087 (0.724–0.921)	0.001	0.776 (0.708–0.851)	< 0.001	0.821 (0.724–0.932)	0.002
Yes	Reference		Reference		Reference		Reference	
Laterality								
Left	Reference		Reference					
Right	1.011 (0.926–1.105)	0.801	1.010 (0.895–1.140)	0.873				
Primary site								
Upper	1.118 (1.017–1.229)	0.020	1.159 (1.017–1.321)	0.027				
Middle	1.061 (0.860–1.308)	0.582	1.126 (0.849–1.495)	0.410				
Lower	Reference		Reference					
Histopathology								
ADC	1.356 (1.221–1.505)	< 0.001	1.425 (1.237–1.641)	< 0.001	1.203 (1.082–1.337)	0.001	1.241 (1.075–1.432)	0.003
SCC	1.870 (1.667–2.097)	< 0.001	1.649 (1.402–1.939)	< 0.001	1.300 (1.152–1.467)	< 0.001	1.112 (0.940–1.317)	0.216
Others	Reference		Reference		Reference		Reference	
Grade								
I	Reference		Reference		Reference		Reference	
II	2.065 (1.803–2.365)	< 0.001	2.370 (1.942–2.891)	< 0.001	1.745 (1.517–2.008)	< 0.001	2.123 (1.731–2.604)	< 0.001
III–IV	2.648 (2.307–3.040)	< 0.001	3.321 (2.718–4.057)	< 0.001	2.180 (1.887–2.520)	< 0.001	2.967 (2.410–3.652)	< 0.001

OS, overall survival; LCSS, lung cancer-specific survival; HR, hazard ratio; CI, confidence interval; ADC, adenocarcinoma; SCC, squamous cell carcino

Multivariate analyses were performed using the Cox regression model and included surgical procedure, age, sex, race, marital status, histopathology result, and tumor grade. The results revealed that surgical procedure, age, sex, race, marital status, histopathology result, and tumor grade were independent predictors of OS and LCSS (*P* < 0.05) (Table 2).

### Propensity score matching survival analyses

After 1:2 PSM, all data were complete, and all variables were well-balanced between the groups. The propensity scores before matching were 0.041 ± 0.047 for lobectomy and 0.099 ± 0.103 for segmentectomy (*P* < 0.001), whereas after matching, they were 0.053 ± 0.023 and 0.054 ± 0.023 for lobectomy and segmentectomy, respectively; *P* = 0.855. Finally, 755 patients (lobectomy: 503, segmentectomy: 252) were included in the study. There were no significant differences in baseline characteristics between the matched groups (Table 1). The mean duration of follow-up was 45.87 ± 34.64 (lobectomy: 44.95 ± 33.38, segmentectomy: 47.72 ± 37.03) months; *P* = 0.300. The median OS was 68 (95% CI 59.99–76.01) months for patients receiving lobectomies, *vs.* 68 (95% CI 56.39–79.61) months for patients receiving segmentectomies. For patients receiving lobectomies and those receiving segmentectomies the 1-, 3-, 5-, and 10-year OS rates were 88.2**,** 70.5, 54.1, and 26.1%; and 90.8, 72.8, 55.2, and 30.7%, respectively. However, the OS (HR 0.914, 95% CI 0.730–1.444; *P* = 0.434) and LCSS (HR 0.920; 95% CI 0.677–1.249; *P* = 0.593) were not significantly different between the lobectomy and segmentectomy groups after matching (Fig. 2c, d).

### Subgroup analyses of the matched groups

**Fig. 3:**
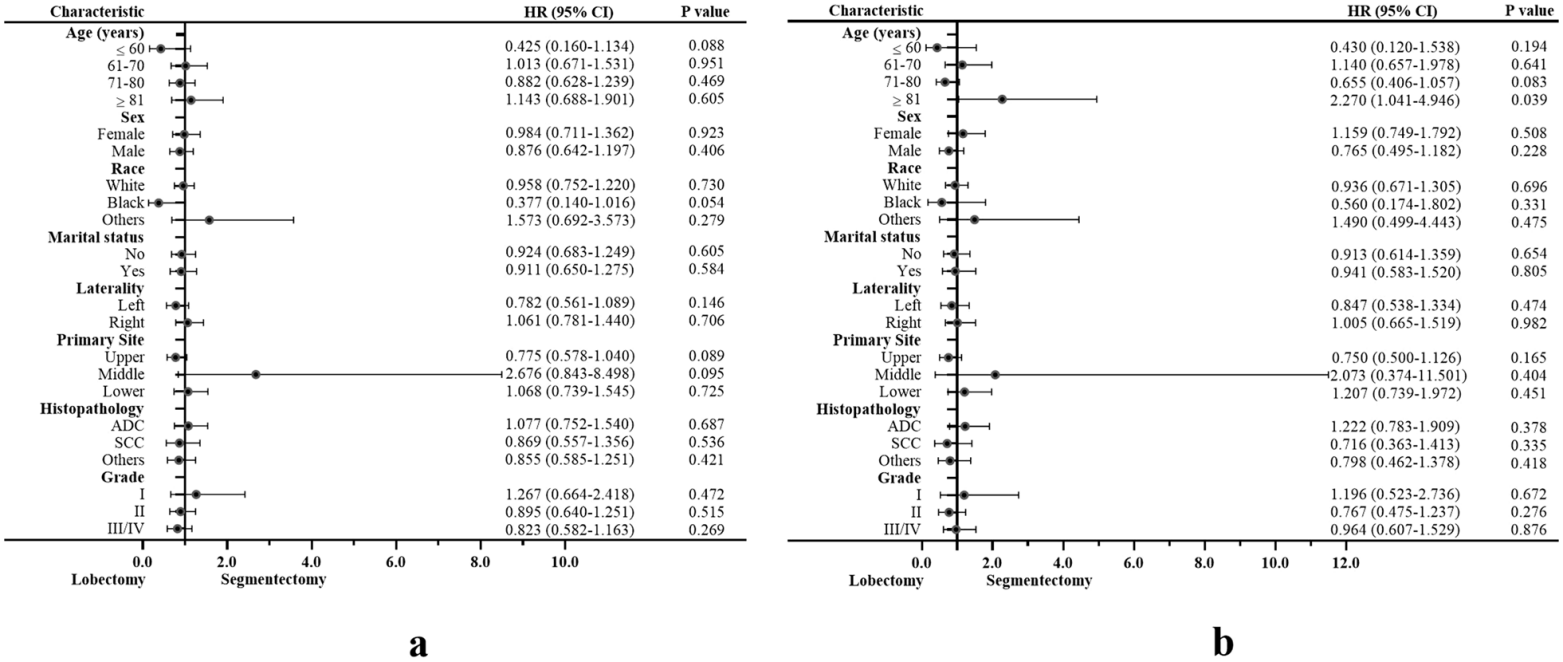
Forest plot of individual hazard ratios for overall survival (**a**) and lung cancer-specific survival (**b**) in patients with lobectomy vs. segmentectomy. Abbreviations: *ADC*, adenocarcinoma; *SCC*, squamous cell carcinoma

Univariate analyses to identify possible prognostic factors after matching revealed statistically significant correlations between OS and LCSS for age, sex, histopathology, and tumor grade (*P* < 0.05). The multivariate analyses also revealed that age and tumor grade were independent predictors of OS and LCSS (*P* < 0.05) (Table 3). The subsequent multivariable Cox regression model showed that younger age and lower tumor grades were significant independent positive prognostic factors for OS. Older age and higher tumor grades (both *P* < 0.05) were significant independent negative prognostic factors for LCSS. The forest plot of individual hazard ratios for overall survival and lung cancer-specific survival in patients with lobectomy vs. segmentectomy (Fig. 3).

**Table 3:** Univariate and multivariate analyses of OS and LCSS after propensity score matching

Characteristic	Univariate analysis (matching)				Multivariate analyses (matching)			
	OS		LCSS		OS		LCSS	
	HR (95% CI)	*P* value	HR (95% CI)	*P* value	HR (95% CI)	*P* value	HR (95% CI)	*P* value
Surgical procedure								
Lobectomy	Reference		Reference					
Segmentectomy	0.914 (0.730–1.444)	0.434	0.920 (0.677–1.249)	0.593				
Age (years)								
≤ 60	Reference		Reference		Reference		Reference	
61–70	2.231 (1.456–3.420)	< 0.001	2.016 (1.152–3.527)	0.014	2.012 (1.310–3.089)	0.001	1.804 (1.028–3.164)	0.040
71–80	3.530 (2.345–5.314)	< 0.001	3.265 (1.918–5.558)	< 0.001	3.169 (2.101–4.780)	< 0.001	2.966 (1.738–5.062)	< 0.001
≥ 81	4.836 (3.073–7.610)	< 0.001	3.119 (1.670–5.827)	< 0.001	4.780 (3.030–7.540)	< 0.001	3.078 (1.643–5.765)	< 0.001
Sex								
Female	Reference		Reference		Reference		Reference	
Male	1.419 (1.149–1.753)	0.001	1.408 (1.056–1.876)	0.020	1.212 (0.979–1.501)	0.078	1.228 (0.918–1.642)	0.166
Race								
White	1.116 (0.730–1.705)	0.613	1.074 (0.610–1.891)	0.804				
Black	0.816 (0.460–1.445)	0.485	0.854 (0.401–1.818)	0.683				
Others	Reference		Reference					
Marital status								
No	0.892 (0.722–1.102)	0.288	1.064 (0.795–1.424)	0.677				
Yes	Reference		Reference					
Laterality								
Left	Reference		Reference					
Right	1.117 (0.905–1.379)	0.301	1.191 (0.894–1.587)	0.231				
Primary site								
Upper	1.221 (0.978–1.525)	0.077	1.189 (0.880–1.607)	0.259				
Middle	1.076 (0.607–1.906)	0.803	1.012 (0.464–2.204)	0.976				
Lower	Reference		Reference					
Histopathology								
ADC	1.298 (1.012–1.663)	0.040	1.589 (1.138–2.218)	0.007	1.194 (0.927–1.537)	0.169	1.456 (1.037–2.047)	0.030
SCC	1.619 (1.238–2.119)	< 0.001	1.502 (1.021–2.209)	0.039	1.248 (0.944–1.650)	0.120	1.166 (0.781–1.740)	0.453
Others	Reference		Reference		Reference		Reference	
Grade								
I	Reference		Reference		Reference		Reference	
II	1.881 (1.325–2.670)	< 0.001	1.612 (1.023–2.538)	0.039	1.749 (1.216–2.516)	0.003	1.430 (0.894–2.289)	0.136
III–IV	2.567 (1.803–3.653)	< 0.001	2.218 (1.405–3.502)	0.001	2.267 (1.570–3.274)	< 0.001	1.957 (1.216–3.147)	0.006

OS, overall survival; LCSS, lung cancer-specific survival; HR, hazard ratio; CI, confidence interval; ADC, adenocarcinoma; SCC, squamous cell carcinoma

## Discussion

Surgery is the first-line treatment for NSCLC, and it is also the only method proven to cure lung cancer. Radical lobectomy resection remains the preferred treatment for early NSCLC. With the popularization of low-dose computed tomography for lung cancer screening, the detection rate for patients with early lung cancer has improved [8], and segmentectomy is being more widely used as a surgical treatment. Recent studies revealed that patients with NSCLC who underwent segmentectomies for lesions < 2 cm obtained similar oncologic effects compared with those that received lobectomies [9–12]. Patients receiving segmentectomies also retained more lung function [13, 14]. The NCCN guidelines indicate that the standard recommendation for the treatment of early NSCLC patients is anatomic pulmonary resection. These guidelines further state that sublobar resection (i.e., segmentectomy or wedge resection) can be appropriate in select patients with the following indications if technical conditions permit and do not increase the surgical risk: (1) Poor pulmonary reserve or another major comorbidity that contraindicates lobectomy; (2) Peripheral nodules ≤ 2 cm with at least one of the following, pure ADC in situ (AIS) on histopathology, nodules with ≥ 50% ground-glass appearance on CT scans, and radiologic surveillance confirming an extended doubling time (≥ 400 days) [15].

However, the more appropriate surgical treatment for patients with early-stage NSCLC remains debatable [16]. As a minimally invasive procedure, lobectomies do not retain as much normal lung tissue as possible under the premise of ensuring efficacy [17]. Segmentectomy involves more anatomic complexity and variation than lobectomy, and requires precise lesion positioning during surgery and the identification of lung boundaries [18–20]. Therefore technically, segmentectomy is a more difficult and demanding procedure than lobectomy. However, TS is an influencing factor for early NSCLC prognoses [21]. The results of an ongoing Randomized Controlled Trial (JCOG0802), have not reached a conclusion [22]. However, Dai et al. [23] reported that patients with NSCLC with tumors < 1 cm or between 1 and 2 cm receiving segmentectomies had worse OS and LCSS than patients receiving lobectomies. Veluswamy et al. [24] demonstrated that in patients with ADC tumors < 2 cm, the OS and LCSS after segmentectomy were similar to those of lobectomy. For SCCs, the OS and LCSS after segmentectomy were inferior to those of lobectomy. In our study, regarding the surgical procedure, we observed that before PSM, for OS and CSS, lobectomy achieved better outcomes than segmentectomy in early T (> 2 cm and ≤ 3 cm) N0N0 NSCLC lung cancer. However, similar to recent studies [25], after PSM, our results revealed no significant differences in patient survival between those receiving lobectomy vs. segmentectomy. Our research shows that for the T (> 2 cm and ≤ 3 cm) N0N0 stage, segmentectomy and lobectomy achieved the same clinical benefit and prognoses regarding OS and LCSS in patients with NSCLC. Nevertheless, further studies are required focusing on the solid component effects and pathologic tumor types with respect to segmentectomies. In addition, age has been identified as a prognostic factor for OS and LCSS. With expanded cancer screening and the wide use of low-dose computed tomography, more patients are being diagnosed at an earlier age [26]. Recently, researchers suggested that postoperative complications are similar between the two procedures [27]. Therefore, whether segmentectomy can be safely and effectively applied in early NSCLC requires further research. This study provides a clinical basis for further investigation by the JCOG0802/WJOG4607L, JCOG1211, JCOG0804/WJOG4507L clinical trials [13, 28].

Compared with lobectomy, a major advantage of segmentectomy is the preservation of lung function. In theory, segmentectomy remove less lung tissue; however, preservation depends on the residual lung function after surgery. Therefore, the impact of the two procedures on lung function remains uncertain [29]. Harada et al. [30] reported that segmentectomy preserved more lung function than lobectomy, with segmentectomy exhibiting less lung function losses after surgery. Gu et al. [31] indicated that segmentectomies could help minimize forced vital capacity (FVC) loss, but not forced expiratory volume in 1 min (FEV_1_) or the diffusing capacity of the lung for carbon monoxide (DLCO). For a single lung segment resected after segmentectomy, the loss of lung function is twice that after lobectomy. However, for multiple pulmonary nodules, segmentectomy can potentially reduce the loss of lung function even further. Waller et al. [32] reported that for multiple primary lung cancer types, segmentectomy is recommended, and lung resection should be avoided; segmentectomy can also allow the performance of future lobectomies. Therefore, compared with lobectomy, segmentectomy could have more advantages for the retention of lung function over the short-term. The advantages of long-term lung function retention after segmentectomy requires further exploration. In the current study, we were unable to compare the differences in lung function concerning long-term survival after lobectomy *vs*. segmentectomy because of database limitations.

### Limitations

As our data were collected from the SEER database, some biases and errors exist despite our PSM analysis. Limitations included (1) lack of detailed information regarding pre-, peri-, and postoperative patient details and outcomes; (2) none or unknown variables (such as tumor component) were grouped together, which could have led to data biases; (3) the 8th American Joint Committee on Cancer staging system was used, which possessed some inconsistencies in the data transformation process compared with earlier versions; and (4) the SEER database lacked information on imaging, smoking history, tumor markers, previous target therapy or immunotherapy, as well as several other parameters; therefore, our study could not address the impact of these factors on patient prognoses after segmentectomy or lobectomy, although they could have played significant roles**.**

## Conclusions

Patients with stage T (> 2 cm and ≤ 3 cm) N0M0 NSCLC undergoing segmentectomy can obtain OS and LCSS similar to those undergoing lobectomy. Further studies focusing on the solid component effects and pathologic tumor types regarding segmentectomies are required. Additional long-term survival and outcome analyses should be conducted with larger cohorts to provide more robust data.

## Data Availability

The datasets supporting the conclusions of this article are included within the article.

## Abbreviations

NSCLC: Non-small cell lung cancer
SEER: Surveillance, Epidemiology, and End Results
CSS: Cancer-specific survival
CT: Computed tomography
PSM: Propensity score matching
OS: Overall survival
LCSS: Lung cancer-specific survival
SDs: Standard deviations
HR: Hazard ratio
CI: Confidence interval
AJCC: American Joint Committee on Cancer
ADC: Adenocarcinoma
SCC: Squamous cell carcinoma
PASS: Power analysis and sample size
NCCN: National Comprehensive Cancer Network
RCT: Randomized Controlled Trial
LDCT: Low-dose computed tomography
FVC: Forced vital capacity
FEV_1_: Forced expiratory volume in 1 min
DLCO: Carbon monoxide lung diffusion capacity
